# Hierarchical hyper-branched titania nanorods with tuneable selectivity for CO_2_ photoreduction†

**DOI:** 10.1039/d1ra05414g

**Published:** 2021-09-28

**Authors:** Gavrielides Stelios, Jeannie Z. Y. Tan, M. Mercedes Maroto-Valer

**Affiliations:** Research Centre of Carbon Solutions (RCCS), School of Engineering & Physical Sciences, Heriot–Watt University Edinburgh UK J.Tan@hw.ac.uk M.Maroto-Valer@hw.ac.uk

## Abstract

Utilising captured CO_2_ and converting it into solar fuels can be extremely beneficial in reducing the constantly rising CO_2_ concentration in the atmosphere while simultaneously addressing energy crisis issues. Hence, many researchers have focused their work on the CO_2_ photoreduction reaction for the last 4 decades. Herein, the titania hyper-branched nanorod (HBN) thin films, with a novel hierarchical dendritic morphology, revealed enhanced CO_2_ photoreduction performance. The HBNs exhibited enhanced photogenerated charge production (66%), in comparison with P25 (39%), due to the unique hyper-branched morphology. Furthermore, the proposed HBN thin films exhibited a high degree of control over the product selectivity, by undergoing a facile phase-altering treatment. The selectivity was shifted from 91% towards CO, to 67% towards CH_4_. Additionally, the HBN samples showed the potential to surpass the conversion rates of the benchmark P25 TiO_2_ in both CO and CH_4_ production. To further enhance the selectivity and overall performance of the HBNs, RuO_2_ was incorporated into the synthesis, which enhanced the CH_4_ selectivity from 67% to 74%; whereas the incorporation of CuO revealed a selectivity profile comparative to P25.

## Abstract 2

The use of hierarchical 1–3D Hyper-Branched Nanorods (HBNs) is examined as a photocatalyst for CO_2_ photoreduction, utilising a facile protonation treatment able to tune the selectivity of CO_2_ photoreduction products between (91%) CO to (67%) CH_4_.

## Graphical abstract

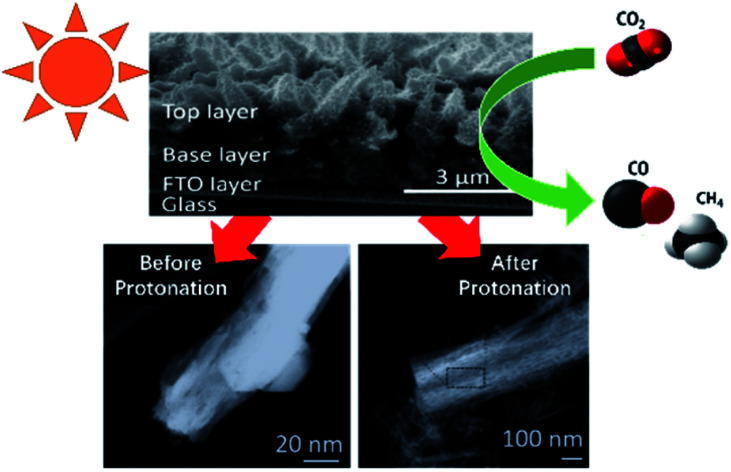

## Introduction

CO_2_ photoreduction is one of the promising carbon neutral energy sources, which has the potential to produce solar fuels, such as CH_4_ and CO, by using water as an electron donor and light as the input of energy.^1,2^ However, CO_2_ photoreduction is a thermodynamically unfavourable process, which requires a large energy input to stimulate the occurrence of the reduction reaction.^3^ The main challenge for CO_2_ photoreduction is the limited light energy absorbance in addition to the high stability of the CO_2_ molecule, and thus high energy input is required for this conversion (1498 kJ mol^−1^).^4^ Consequently, the fabrication of highly efficient photocatalysts becomes a very important task.

TiO_2_ is the most extensively used photocatalyst for photocatalysis as well as CO_2_ photoreduction, accounting for roughly 40% of the publications on CO_2_ photoreduction.^5–11^ The main advantages of TiO_2_ are its chemical and thermal stability, high charge transfer potential, abundance, non-toxicity and cost.^12–14^ TiO_2_ has a relatively wide band gap, which allows for an effective redox potential for CO_2_ photoreduction, but at the same time limits the range of electromagnetic radiation wavelengths that it can absorb. Hence, the wide band gap and the consequent wavelength absorption limitation is considered the main challenge for TiO_2_.^12,15^ Foreign element doping, semiconductor coupling, and photosensitisation are amongst the most popular methods implemented to expand the light absorbance and improve the photocatalytic performance of TiO_2_.^16^ Despite the fact that morphological alterations wouldn't widen the range of wavelengths that can be absorbed by the photocatalyst, morphology still is an important parameter and it has the potential to improve the amount of light absorbed, increase the surface area and thereby expose more active sites.

The morphological alterations of 3D hierarchical TiO_2_ nanostructures have received considerable attention as they were found to enhance the photocatalytic performance.^17^ This was attributed to the synergistic benefits of their complex structure, which is composed from basic nanoscale building blocks that preserve their unique individual properties (*i.e.*, 0D spheres, 1D nanorods, 2D sheets, *etc*.). Such 3D hierarchical structures, which consists of 1D building blocks that are combined to form 3D super-structures, have been reported to enhance light harvesting due to light scattering effects, increase surface area and improved charge transport.^18–22^

The effects of morphological design for CO_2_ photoreduction is demonstrated herein using hierarchical 1–3D TiO_2_ Hyper-Branched Nanorod (HBNs) grown on Fluorine–Tin Oxide (FTO) conductive glass. The unique morphology of the HBNs microstructures revealed a significant increase of light absorption due to increased number of exposed active sites and light scattering effects. Hence, the fabricated HBNs showed high photocatalytic performance in CH_4_ and CO production under visible light irradiation. Interestingly, the fabricated HBNs demonstrated the ability to shift and increase the product selectivity through a facile protonation treatment. The best performing sample has demonstrated an internal quantum efficiency of *Φ*_CH4_ = 69%.

## Experimental procedure

### Materials & synthesis

Potassium titanium oxide oxalate dihydrate (PTO, ≥98.0%), diethylene glycol (DEG, 99.0%), bis(cyclopentadienyl)ruthenium ((C_5_H_5_)_2_Ru, 98.0%), copper(ii) acetate (Aldrich, 98%), *n*-hexane (C_6_H_14_, 95.0%). Isopropanol (IPA, 99.5%), acetone (>95.0%) and ethanol (99.0%) were procured from Fisher Scientific. All chemicals were used without any further purification. All aqueous solutions were prepared using Milli-Q ultrapure type 1 water (18.2 MΩ.cm) collected from a Millipore system. Fluorine-doped tin oxide (FTO) TEC-15 glass was purchased from Ossila (2.5 cm × 2.5 cm, roughness of 12.5 nm, FTO layer thickness of 200 nm, 83.5% transmission and resistivity of 12–14 Ω cm^−1^).

Before the use of the FTO glass slides, they were cleaned using a solution of H_2_O, IPA, and Acetone in a ratio of 1 : 1 : 1. The glass was submerged into the solution and sonicated for 1 h. The glass was then dried at 75 °C for 30 min.

**Titania hyper-branched nanorods (HBNs BP)** were fabricated using a hydrothermal approach. Similar synthesis has been performed in our previous studies.^23^ PTO was dissolved in a mixture of H_2_O and DEG in a 1 : 7 ratio. The concentration of PTO was 0.05 M. The precursor solution was stirred for 30 min before it was transferred to a 100 mL Teflon-lined autoclave along with the FTO glass. The FTO glass was resting against the Teflon-liner walls with the conductive side facing down at approximately 60°. The hydrothermal synthesis was carried out at 180 °C for 9 h. The autoclave was allowed to cool down to room temperature and the titania HBNs were rinsed several times using Milli-Q, water and ethanol. The rinsed samples were then calcined at atmospheric conditions at 550 °C for 1 h. This sample was depicted as HBNs BP, which stands for “before protonation”.

**Hyper-branched nanorods (HBNs) protonation treatment** was performed after the calcination of the previous step. The as-prepared HBNs BP were allowed to naturally cool down to room temperature and were then submerged in ethanolic HCl (0.04 M) for 3 h and aqueous HCl (0.02 M) for another 3 h, with mild agitation. The HCl solution was replaced with fresh HCl solution every 1 h. Following this procedure, the as-prepared thin-films were rinsed with both ethanol and DI water several times and were submerged in ethanol for another 1 h in mild agitation. They were then dried in the oven at 70 °C overnight before another calcination at atmospheric conditions at 400 °C for 2 h. The resulted sample was depicted as HBNs AP.

**Synthesis of RuO_2_ loaded HBN (RuO_2_-HBNs)** was synthesized under dry nitrogen atmosphere, in a custom-made glove box. Bis(cyclopentadienyl)ruthenium (C_5_H_5_)_2_Ru, was dissolved in *n*-hexane (C_6_H_14_) to achieve a molar concentration of 0.005 M and stirred vigorously at atmospheric temperature for 1.5 h until a clear solution was obtained. The HBNs AP loaded glass-slide was then placed into the Teflon liner resting against its walls at 60° as described before, with the coated surface facing down. The ruthenium precursor liquid was added to the Teflon-liner to cover the entire FTO glass surface (25 mL). The Teflon liner was then transferred into the autoclave and was placed in the oven at 180 °C for 30 h. The Ru–TiO_2_ FTO glass was then rinsed with *n*-hexane and calcined to 400 °C for 10 h with a ramp rate of 10 °C min^−1^.

**Synthesis of CuO loaded TiO_2_ (CuO-HBNs)** was synthesised using copper(ii) acetate, which was dissolved in 50 mL of ethanol at a concentration of 0.005 M. The solution was stirred for 30 minutes. Then 2 mL of NaOH was added dropwise and stirred for another 60 min, obtaining the final solution that was used to fabricate the CuO-HBNs thin film. The Cu precursor solution was transferred to a 100 mL Teflon-lined autoclave along with the HBNs AP loaded FTO glass. The glass was positioned resting against the Teflon-liner walls with the coated side facing down at approximately 60°. The solvothermal treatment was carried out at 150 °C for 5 h. The autoclave was allowed to cool down to room temperature, and the CuO-HBNs slides were rinsed with ethanol and then calcined to 400 °C for 10 h with a ramp rate of 10 °C min^−1^.

### Characterization

Crystallinity and phase identification of the synthesized products were conducted using powder X-ray diffraction XRD (Bruker D8 Advanced diffractometer) equipped with Cu Kα radiation (*λ* = 1.5418 Å) and compared with the ICDD-JCPDS powder diffraction file database. The morphological features of the synthesized samples were examined by a field emission scanning electron microscope (FE-SEM, FEI Quanta 200 F). Further investigation on the morphology at higher magnification and the element composition of the samples was carried out using a high-resolution transmission electron microscope (HR-TEM, FEI Titan Themis 200) equipped with an energy dispersive X-ray spectroscope (EDX) detector operated at 200 kV. The sample for HR-TEM analysis was prepared by gently removing the coating off the FTO glass surface. And then, the powder obtained was suspended in ethanol shaken vigorously for 5 minutes until a cloudy solution was achieved, then a few drops of the solution were placed on a carbon-coated nickel TEM grid and was left to dry in atmospheric conditions. Crystallinity and phase identification of the synthesized products were performed using Raman, which were recorded using a Renishaw *inVia* Raman Microscope with 785 nm excitation source. The diffuse reflectance was measured using a PerkinElmer Lambda 950 UV-vis equipped with an integrating sphere (150 mm) and the band gap energy was estimated using the Kubelka Munk function. X-ray photoelectron spectroscopy (XPS) was performed on Scienta 300 XPS machine incorporating with a rotating Al Kα X-ray source operating at 13 kV × 333 mA (4.33 kW). Electron analysis was performed using a 300 cm radius hemispherical analyser and lens system. The electron counting system consist of a multichannel plate, phosphorescent screen and CCD camera. All multichannel detection counting is done using proprietary Scienta software. The elements present were determined *via* a wide energy range survey scan (200 mW step, 20 ms dwell time, 150 eV pass energy and summed over 3 scans). The high-resolution scans were performed at a similar pass energy (150 eV), but a step size of 20 mV. A dwell time of 533 ms was used and accumulated over 3 scans. The instrument operated at a base pressure of 1 × 10^−9^ mbar; the energy scale is calibrated using the Au 4f, Ag 3d and Cu 2p emission lines, where the half width of the Au 4f_7_ emission line is approximately 1.0 eV. All data analysis and peak fitting were performed using the CaseXPS software. The photon flux was measured by using the spectroradiometer (Apogee-200 from Apogee Instruments), at a set distance of 20 cm from the light source (OmniCure S2000) that was used in the CO_2_ photoreduction reaction. The blank measurement was performed using a blank FTO glass. The quantum efficiency measurement of CO and CH_4_ were calculated using eqn (1) and (2), respectively. The average production value was used for the internal quantum efficiency measurement of each product.1
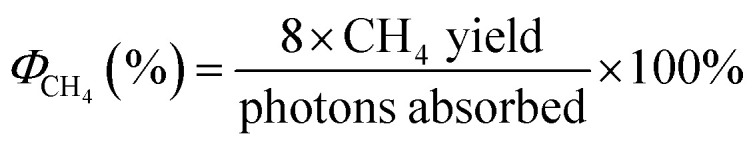
2
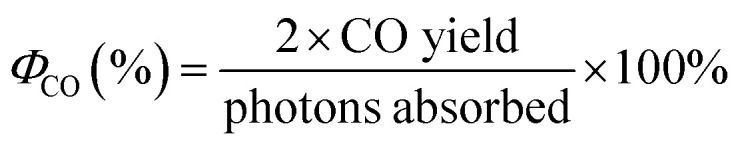


### Photocatalytic test

**Fig. 1:**
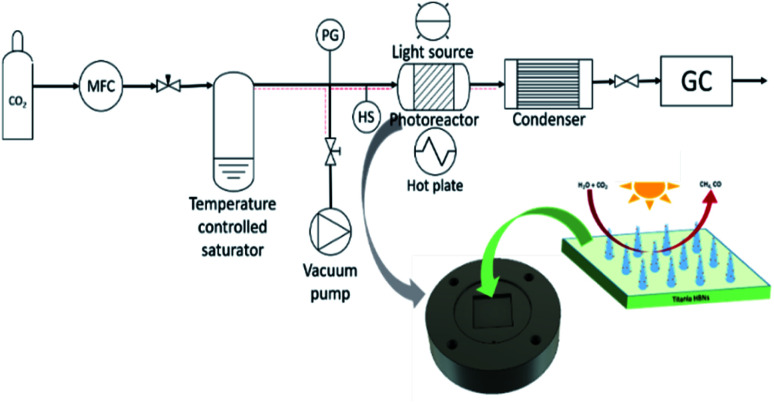
Photoreduction rig set-up diagram (not to scale).

The CO_2_ photoreduction were performed using the experimental set-up as described in previous work.^24^ Briefly, the FTO glass samples were inserted into the cut-out slot of the photoreactor as shown on Fig. 1. Before each photocatalytic test, a purge and equilibration regime was followed, where the system was placed under vacuum (−100 kPa) and then the vacuum was released with CO_2_ (99.995%) until it reached 100 kPa of pressure. The pressure was then released through the injection port of the gas chromatograph (GC, Agilent, Model 7890B series). This process was repeated three times and on the final repetition the system was left with a CO_2_ flow rate of 0.35 mL min^−1^ passing through a temperature-controlled saturator for at least 16 h (overnight) to allow the system to equilibrate. Relative humidity (±1.8% RH) was measured using an inline Sensirion SHT75 humidity sensor embedded (MG Chemicals 832HD) nested into a Swagelok 1/4′′ T-piece. The temperature of the photoreactor (40 °C) was controlled using a hot plate and the surface of the photocatalyst measured using a Radley's pyrometer (±2.0 °C). An OmniCure S2000 light source (300–600 nm wavelength) was placed 30 mm above the surface of the investigated sample. Irradiance (150 mW cm^−2^) at the end of the fiber optic light guide was measured before each experiment using an OmniCure R2000 radiometer (±5%). An inline GC with a Hayesep Q column (1.5 m, 1/16 inch OD, 1 mm OD), Molecular Sieve 13X (1.2 m, 1/16-inch OD, 1 mm ID), thermal conductivity detector (TCD), nickel catalyzed mechanizer, and flame ionization detector (FID) was used to analyze the output of the photoreactor every four min. The GC was calibrated using 1000 ppm calibration gas (H_2_, CO, O_2_ and CH_4_ in Ar balance). It was then further diluted with Ar (99.995%) using mass flow controllers to 17.04, 4.62 and 1 ppm using the FID detector for CH_4_ and CO, respectively, and 69.49, 34.72 and 17.04 ppm using the TCD detector for H_2_ and O_2_.

Control experiments were carried using all the fabricated samples to investigate the genuine production of carbonaceous products from CO_2_.

(1) CO_2_ was replaced with N_2_;

(2) Light source was turned off.

Other experimenting parameters were unchanged.

## Results and discussion

**Fig. 2:**
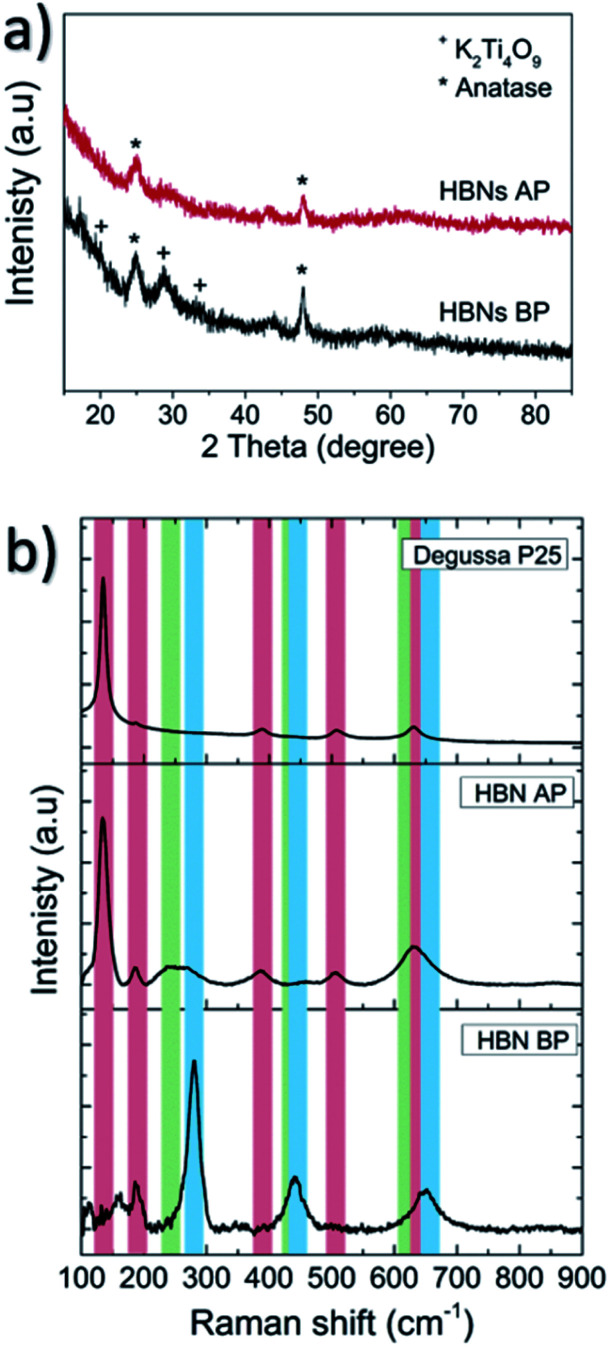
X-ray diffraction pattern for the as prepared HBNs BP and HBNs AP (a), Raman spectra of the fabricated samples with potassium titanates (blue), anatase (red) and rutile (green) components (b).

The titania HBNs were fabricated using a facile hydrothermal treatment on conductive FTO glass slides. The XRD pattern of HBNs BP evidenced the presence of anatase and K_2_Ti_4_O_9_ (Fig. 2a). After the protonation treatment, K_2_Ti_4_O_9_ was removed, leaving only anatase phase within the sample HBNs AP. Due to the weak signal in XRD, Raman spectroscopy was utilised to investigate the crystallinity and crystal phase of the samples (Fig. 2b). Raman signal responds to spatial order, associated with crystalline structures, with a sharp peak and narrow bands, whereas amorphous solids and their lack of spatial order in the crystal lattice translates to broad peak signals.^25^ The as-prepared HBNs BP sample exhibited mainly potassium titanate features shown at 188, 279, 441 and 652 cm^−1^.^26^ Meanwhile, a weak signal of the anatase phase feature was observed at 143, 395, 517, and 638 cm^−1^ associated with the E_g_, B_1g_, A_1g_, and E_g_ vibrations, respectively.^23^ Potassium titanate was the dominant phase and the relative ratio of potassium titanate to anatase was estimated at 7 : 1.

The HBNs BP was treated with a facile protonation treatment forming HBNs AP. As a result, a phase shift was observed, in which the anatase phase (*i.e.*, 143, 395, 517, and 638 cm^−1^, Fig. 2) was shown the dominant phase. The relative ratio of potassium titanate to anatase was reduced to 1 : 10. Additionally, traces of rutile phase were spotted at 442 and 631 cm^−1^, attributing to the use of HCl acid.^26^ Degussa P25, which is used as the benchmark sample in this study was also characterised as shown in Fig. 2.

**Fig. 3:**
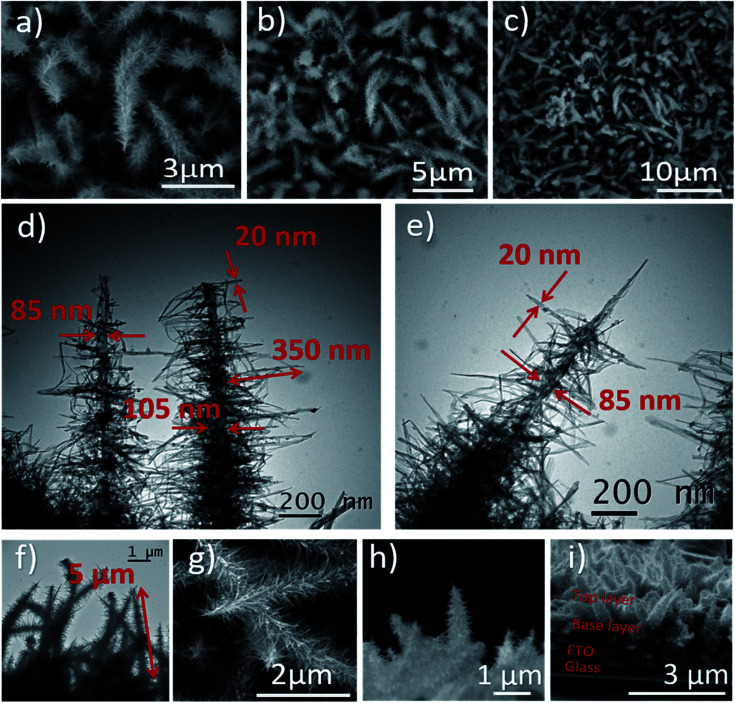
The HBNs BP sample under SEM (a–c) and TEM (d–f). The top view (g) and cross-section (h and i) of HBNs AP sample under SEM.

The as-prepared HBNs samples showed homogeneous and full coverage of coating on the surface of the FTO glass (Fig. 3a–c). A dendritic microstructure (3–5 μm in height) was revealed, decorated with numerous nano-branches growing along the dendrites. The growth orientation was generally upwards with a chaotic directionality of the nano-branches. The width and length of the nano-branches were ∼5–20 and 50–350 nm, respectively (Fig. 3d–g).^23,27,28^

**Fig. 4:**
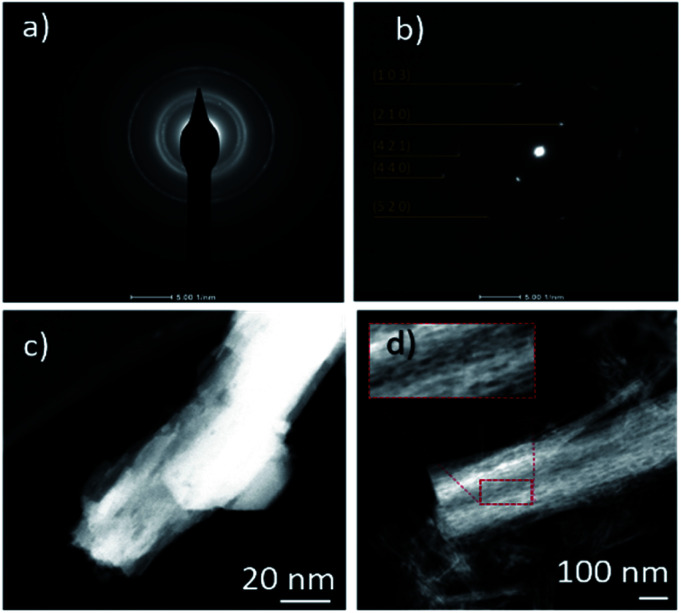
SAED pattern and TEM images of HBNs BP (a and c, respectively) and HBNs AP (b and d, respectively).

After the protonation treatment, no significant changes were observed in the morphology of the HBNs structure (Fig. 3g) and the thin film remained intact. Prior to the subsequent calcination, the titanate phase of the thin film remained as the dominant phase (Fig. S1†) and exhibited a polycrystalline structure under SAED examination (Fig. 4a). After the calcination, the polycrystalline titanate phase was significantly reduced and a highly crystallised *c*-axis elongated anatase phase emerged (Fig. 4b). As a result of protonation treatment and calcination processes, the formation of pores was observed on the nano-branches and dendrites (Fig. 4c and d). Hence, the mechanism of protonation was proposed. During the protonation treatment, ionic exchange reaction took place, in which potassium titanate converted to protonated-titanate. Then, the subsequent calcination treatment resulted the protonated titanate to re-organise into anatase phase of TiO_2_ as observed in the Raman spectra (Fig. S1†) and the re-organisation created the observed porosity (Fig. 4d).^26,29^

**Fig. 5:**
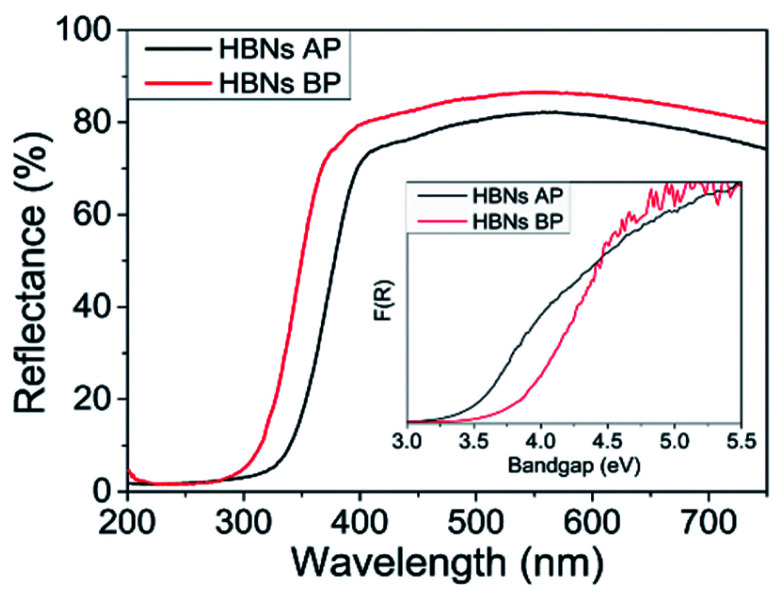
Diffuse reflectance spectra with a Kubelka–Munk inset for band gap calculation.

The optical properties of the HBNs were analysed using UV-vis and spectroradiometry. The HBNs BP sample showed higher reflectance compared to the HBN AP, indicating the latter possessed higher optical properties than the former (Fig. 5). Moreover, the cut-off wavelength of the HBNs AP was longer than HBNs BP, implying a band gap narrowing after the protonation treatment. The band gap of the HBNs BP was estimated at 3.7 eV; whereas after protonation treatment, the band gap was reduced to 3.4 eV. This was attributed to the shift from titanates to anatase phase. The photon flux of HBNs AP increased from 39.8 (*i.e.*, HBNs BP sample) to 48.2 μmol m^−2^ under irradiation of visible light in the range of 300–600 nm. The increase of photon flux could be explained by the band gap narrowing as shown in the UV-vis results, in which more photogenerated charges were excited to the conduction band and available for the CO_2_ photoreduction reaction. Meanwhile, the benchmark P25 showed a photon flux of 28.6 μmol m^−2^. Hence, the effective photon flux for the photocatalytic reaction obtained from the HBNs BP, HBNs AP and P25 samples were 54, 66 and 39%, respectively. As expected, the amount of light absorbed by the HBNs microstructures was significantly higher than P25 due to the unique morphology of the HBNs even though P25 possessed a narrower band gap.

The CO_2_ photoreduction reaction was performed using a custom-made reactor designed for the FTO glass and was connected and analysed using GC (Fig. 1).^24,30,31^ The HBNs BP exhibited a cumulative conversion of CO at 7.9 μmol g_cat_^−1^ h^−1^. On the other hand, it exhibited a very low selectivity towards CH_4_ (9%) and produced only 0.4 μmol g_cat_^−1^ h^−1^ of CH_4_ in contrast to 6.9 μmol g_cat_^−1^ h^−1^ for P25. Although the overall conversion of HBNs BP was the lowest among the samples, the superior selectivity towards CO resulted the highest production of CO. HBNs AP displayed a selectivity shift towards CH_4_ (67%), producing the highest cumulative conversion of CH_4_ (8.7 μmol g_cat_^−1^ h^−1^, Table 1) surpassing both the P25 and HBNs BP sample. Therefore, the HBNs had shown great flexibility in adjusting the product selectivity by treating the sample using a facile protonation step. It is worth noting that the HBNs AP with dominating anatase phase had shown significantly higher photocatalytic activity compared to the titanate rich HBNs BP. This was attributed to the higher charge transfer potential associated with the anatase phase of TiO_2_ as well as the porosity formed which provides easy access to the surface of the material.^32^ To the best of the authors knowledge, the utilisation of a simple protonation step to alter the selectivity of the HBN material for CO_2_ photoreduction has not been reported in the literature.

**Table 1:** Results of CO_2_ photoreduction for HBNs BP, HBNs AP Degussa P25, RuO_2_-HBNs and CuO-HBNs. Unprocessed GC data is available in Fig. S4. Selectivity calculations take into consideration only CH_4_ and CO production values

Sample name	CH_4_ selectivity (%)	CH_4_ cumulative production (μmol g_cat_^−1^ h^−1^)	CO selectivity (%)	CO cumulative production (μmol g_cat_^−1^ h^−1^)
HBNs BP	9	0.4	91	7.9
HBNs AP	67	8.7	33	4.3
Degussa P25	56	6.9	44	5.1
RuO_2_-HBNs	74	5.2	26	1.8
CuO-HBNs	53	2.5	47	2.2

To further investigate the potential of the already modified selectivity and performance of the as-prepared photocatalyst, the HBNs microstructures were loaded with RuO_2_ and CuO, respectively and the photoreduction results are shown in Table 1. The best performing HBNs AP sample (*i.e.*, in terms of cumulative overall conversion), was employed for the synthesis of the RuO_2_-HBNs and CuO-HBNs samples. HBNs AP, RuO_2_-HBNs and CuO-HBNs were examined under Raman and revealed very similar peaks of predominantly anatase, weak signals of potassium titanate and traces of rutile.

However, no Ru or Cu related peaks could be identified (Fig. S2†). This could be attributed to the low concentration of the RuO_2_ and CuO as well as highly dispersed clusters of these foreign elements.^23,33^ The microstructure of RuO_2_-HBNs and CuO-HBNs did not show significant changes after the loading of RuO_2_ and CuO.

**Fig. 6:**
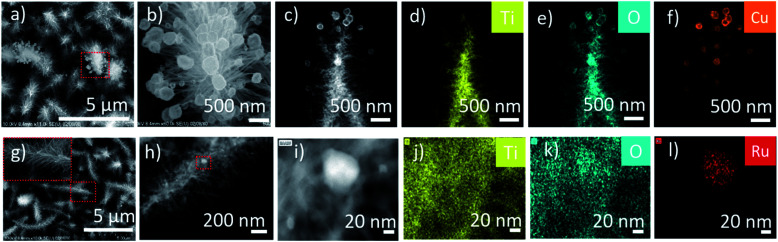
Microscopy morphology investigation and elemental mapping EDX. (a and b) SEM imaging for CuO-HBNs, (c–f) EDX analysis for CuO-HBNs. (g) SEM imaging with zoomed inset for RuO_2_-HBNs, (h) TEM imaging for RuO_2_-HBNs, (i–l) EDX analysis for RuO_2_-HBNs.

The CuO-HBNs samples revealed some dangling nanoparticles (50–150 nm) attached to the tips of the nano-branches of the HBNs (Fig. 6a and b). These particles were evidenced as Cu nanoparticles under the elemental analysis of HR-TEM (Fig. 6c–f). Upon closer inspection of RuO_2_-HBNs under HR-TEM, some nanoparticles (50–60 nm) were found loaded on the HBNs structures (Fig. 6g–i). Elemental analysis was performed to identify the elemental composition of the observed nanoparticles on the sample. Ru element was spotted and identified on the HBNs while Ti and O were identified as the dominant elements of the HBN structure (Fig. 6i–l).

**Fig. 7:**
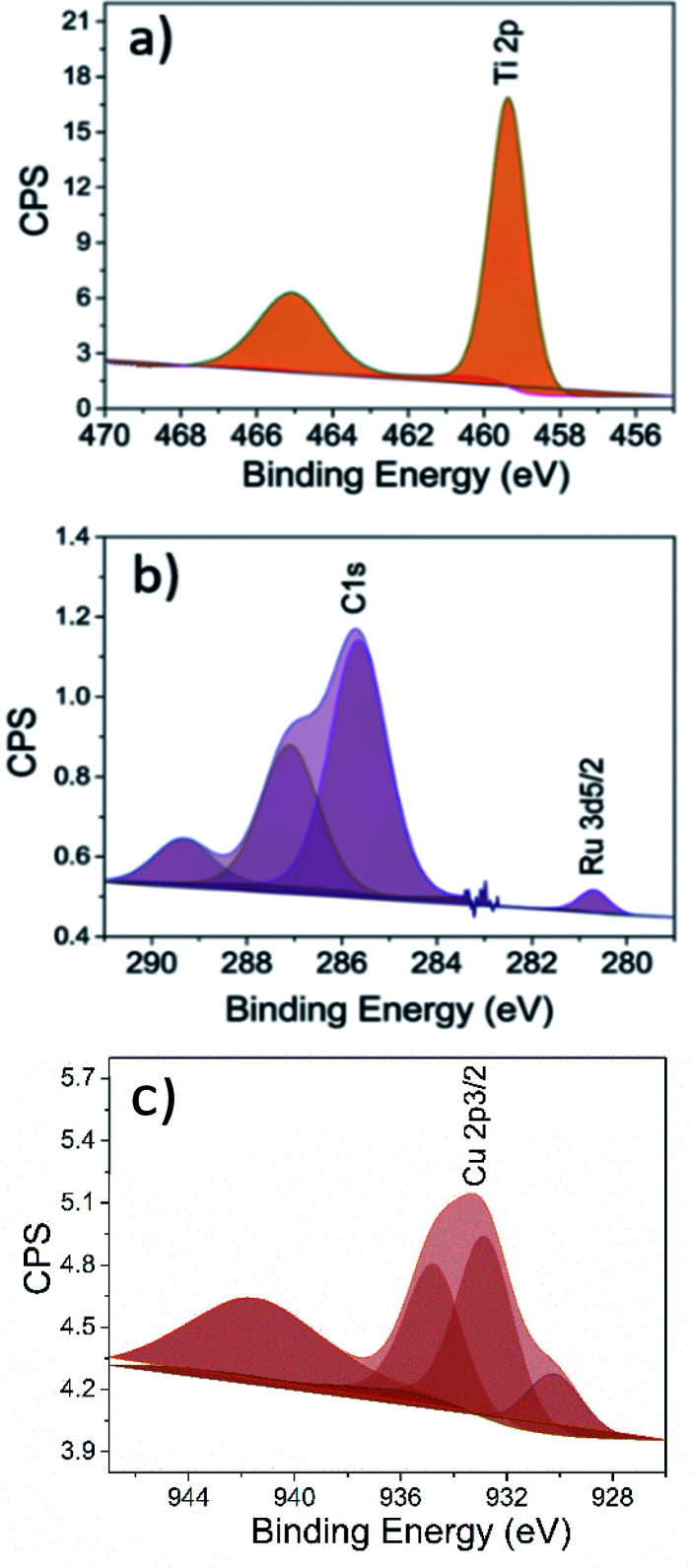
XPS analysis, magnification of the peaks (a) Ti 2p of HBNs AP, (b) Ru 3d_5/2_ of RuO_2_-HBNs and (c) Cu 2p_3/2_ of CuO-HBNs samples.

Further insight regarding elemental composition was acquired utilising XPS. The XPS results showed that the loaded Cu and Ru was 3 and 1 at%, respectively. The Ru 3d_5/2_ peak was found at 280.7 eV, which is associated to Ru^4+^, evidencing the presence of RuO_2_ nano-particles on the RuO_2_-HBNs sample (Fig. 7b).^34,35^ Regarding the CuO-HBNs sample, the Cu 2p_3/2_ peak emerged at 932.9 eV and was attributed to Cu^2+^, which led to the conclusion that the observed dangling particles were CuO nanoparticles (Fig. 7c). The Ti 2p with 5.7 eV of spacing between Ti 2p_1/2_ and 2p_2/3_ evidenced the presence of TiO_2_. The Ti 2p, O 1s as well as the adventitious carbon C 1s peaks were found in all samples as expected under X-ray photoelectron spectroscopy.^36^ A weak signal of K 2p was observed even after the protonation treatment on the survey scan for both samples, (Fig. S3†) showing some amount of potassium was still present in the sample as it was also confirmed by the Raman investigation.^37^

The optical properties were examined using UV-Vis and spectroradiometer measurements. The RuO_2_-HBNs exhibited a very similar reflectance to the HBN-AP, possibly because of the very low concentration of RuO_2_ on the sample. However, CuO-HBNs has shown increased light absorption in the 350–800 nm range (Fig. 5). The band gap energy of was estimated at 3.4 eV for all the samples as can be seen in the inset of Fig. 5. The spectroradiometer measurements have shown the RuO_2_-HBNs to have slightly reduced photon flux 45 μmol m^−2^ when compared to the HBNs AP 48.2 μmol m^−2^. However, the CuO-HBNs exhibited increased photon flux at 54.2 μmol m^−2^. The results acquired from the GC for RuO_2_-HBNs and CuO-HBNs are shown in Table 1. The RuO_2_-HBNs sample has shown further increase on the CH_4_ selectivity to 74% compared to HBNs AP sample (67%). However, a reduction in conversion yield was observed in RuO_2_-HBNs (*i.e.*, from 8.7 μmol g_cat_^−1^ h^−1^ to 5.2 μmol g_cat_^−1^ h^−1^ in HBNs AP). This was attributed to the reduced photon flux observed under the spectroradiometer measurements when compared with HBNs AP. The improved product selectivity towards CH_4_ as exhibited in RuO_2_-HBNs was attributed to the effective charge separation, and suppressed electron–hole recombination, allowing more photogenerated electrons to participate in CO_2_ photoreduction reaction, consequently amplifying the multi-electron conversion of CH_4_ as opposed to the 2-electron conversion of CO.^38–41^

An altered selectivity profile was observed with the incorporation of CuO for CuO-HBNs where CH_4_ selectivity was decreased compared to HBNs AP. The incorporation of CuO have been reported to improve optical performance and to have the potential to facilitate charge migration.^42^ Despite the improved optical performance observed, the CuO-HBNs exhibited a modest overall conversion even though the loading composition was higher than RuO_2_-HBNs. This was attributed to the CuO functioning as a recombination centre thereby prohibiting the reaction flow most likely due to the agglomerated relatively large ∼300 nm CuO particles observed in Fig. 6a–f. Researchers have previously reported CuO loading acting as a recombination centre resulting in reduced conversion rates.^16,33,43,44^ It has also been reported that excessive CuO loading could result in masking the illuminated TiO_2_ surface therefore reducing conversion rates.^16,35^ Control experiments were conducted as described in the experimental procedure section and no or only trace amount of products was observed on the GC.

## Conclusions

A facile protonation treatment has demonstrated the ability to tune the product selectivity of CO_2_ photoreduction under 300–600 nm of light irradiation. The hierarchical 1–3D nanostructured HBNs had significantly improved the optical properties of TiO_2_, in which the HBNs possessed 66% of photon flux while P25 only showed 39% under 300–600 nm of irradiation. The proposed protonation treatment was able to manipulate the product selectivity of the as-prepared HBNs from 91% towards CO to 67% towards CH_4_. As a result, as-prepared HBNs exhibited the highest production of CO, whereas the HBNs AP had the highest production of CH_4_. Additionally, the overall conversion of HBNs AP was the highest amongst other samples and achieved an overall quantum efficiency of 77%. The RuO_2_ loaded HBNs AP managed to further increase the CH_4_ selectivity to 74%. CuO loaded HBNs showed drastic increased in light absorption with comparative selectivity performance as P25. Overall, the titania HBN thin films have shown to be a high performing and highly versatile nanostructured material with great flexibility in terms of selectivity for CO_2_ photoreduction.

## Author contributions

Stelios Gavrielides: conceptualisation, methodology, data curation, writing the original draft. Jeannie Z. Y. Tan: conceptualisation, methodology, writing-review & editing, supervision. M. Mercedes Maroto-Valer: supervision.

## Conflicts of interest

There are no conflicts to declare.

## Supplementary Material

RA-011-D1RA05414G-s001
