# Supplementary material for: Hierarchical hyper-branched titania nanorods with tuneable selectivity for CO2 photoreduction
Source: RSC Adv. 2021 Sep 28;11(51):32022–9. doi: 10.1039/d1ra05414g (PMC9041433; doi:10.1039/d1ra05414g)
Supplement: RA-011-D1RA05414G-s001 [file RA-011-D1RA05414G-s001.pdf]

Electronic Supplementary Material (ESI) for RSC Advances.
This journal is © The Royal Society of Chemistry 2021

Supplementary information

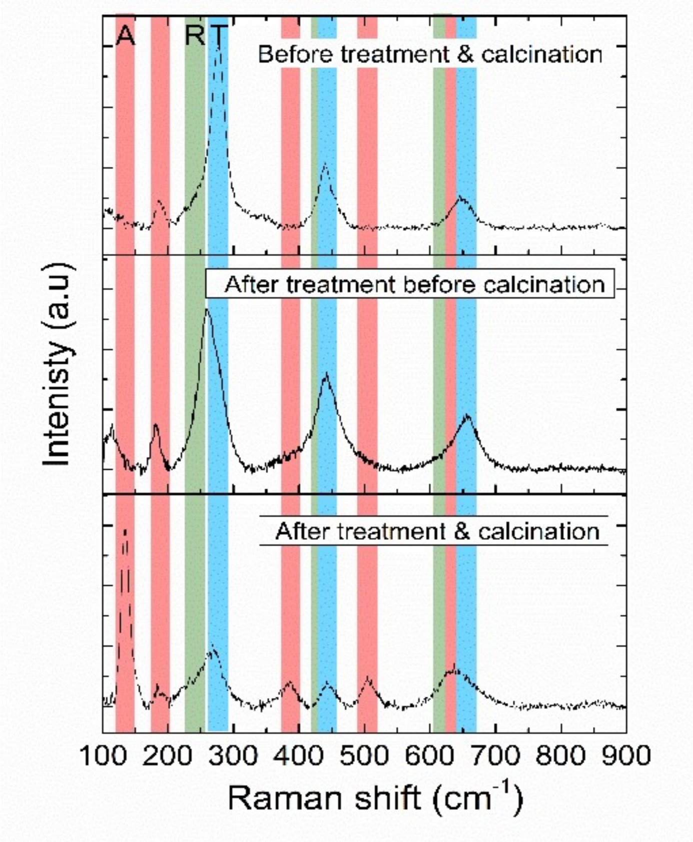

Figure S1 - Raman spectra investigation of the phase altering step from potassium titanates to anatase phase.

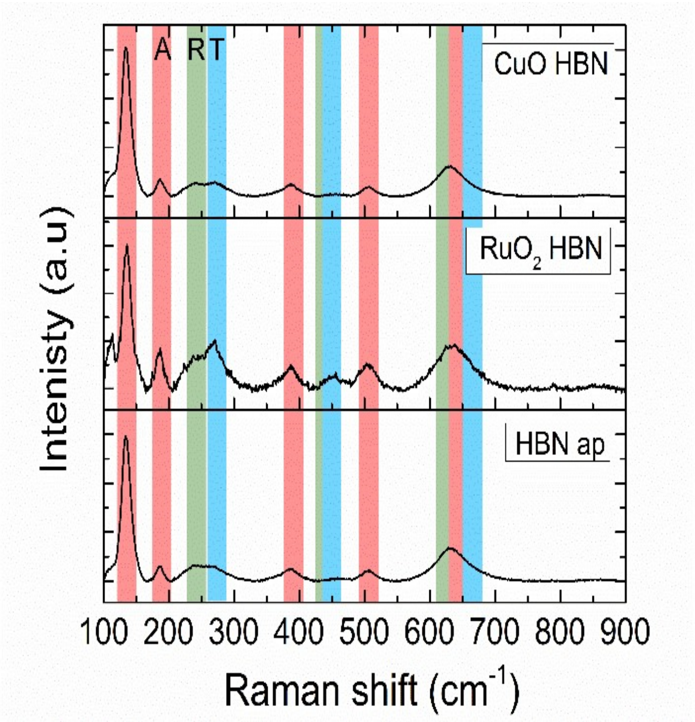

Figure S2 - Raman spectra of the RuO_2 HBN and CuO HBN samples with as-prepared HBN ap. The figure shows very similar spectra with no Ru or Cu related peaks.

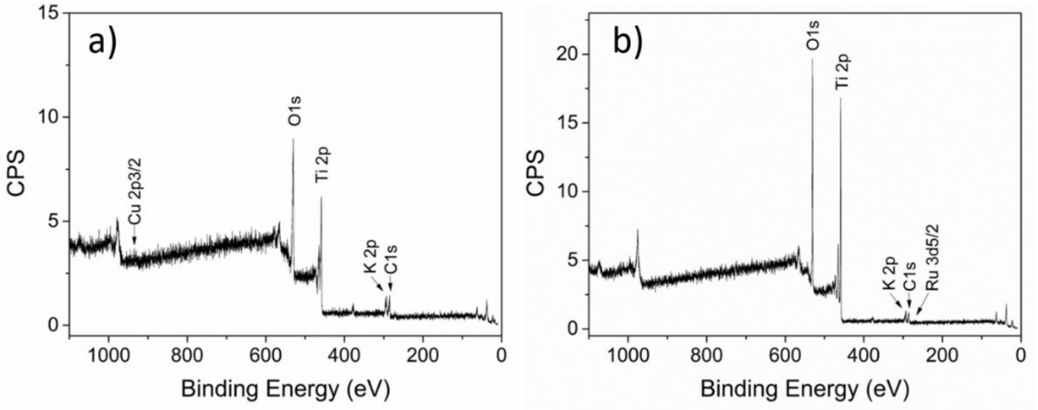

Figure S3 - XPS survey spectra for a) CUO HBN and b) RuO_2 HBN samples with annotated peaks.

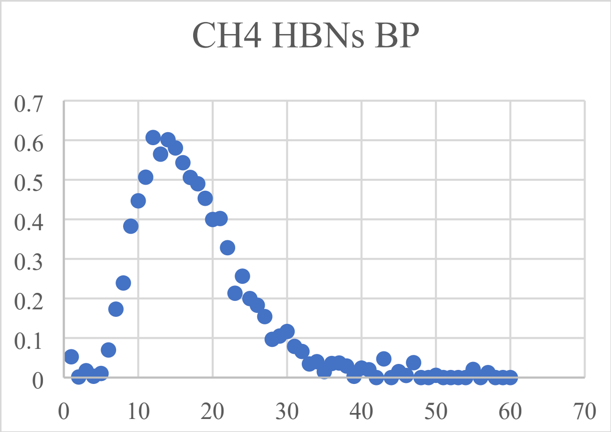
CH4 HBNs BP
0.7 0.6 0.5 0.4 0.3 0.2 0.1 0
0 10 20 30 40 50 60 70

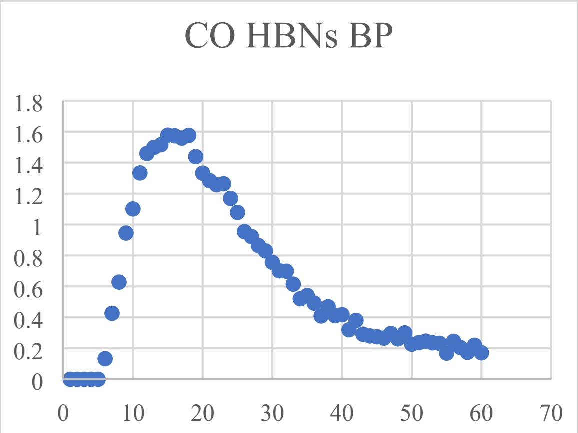
CO HBNs BP
1.8 1.6 1.4 1.2 1 0.8 0.6 0.4 0.2 0
0 10 20 30 40 50 60 70

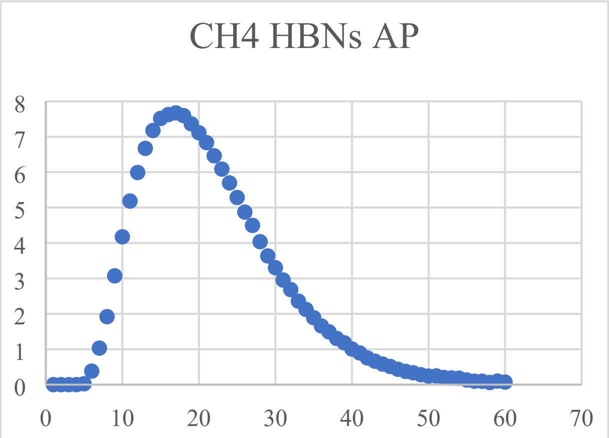
CH4 HBNs AP
8 7 6 5 4 3 2 1 0
0 10 20 30 40 50 60 70

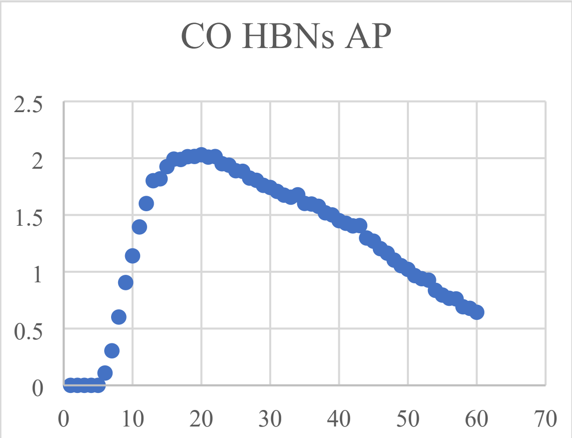
CO HBNs AP
2.5 2 1.5 1 0.5 0
0 10 20 30 40 50 60 70

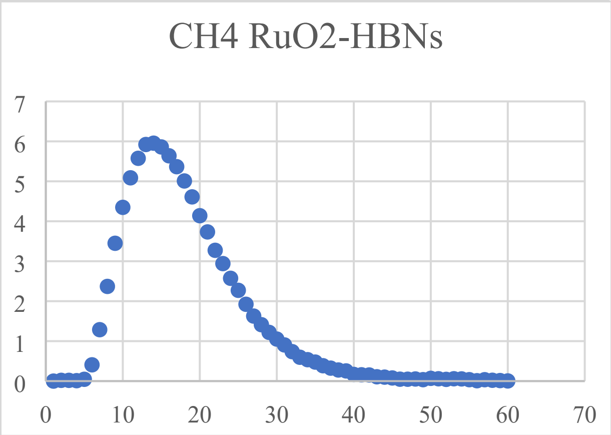
CH4 RuO2-HBNs
7 6 5 4 3 2 1 0
0 10 20 30 40 50 60 70

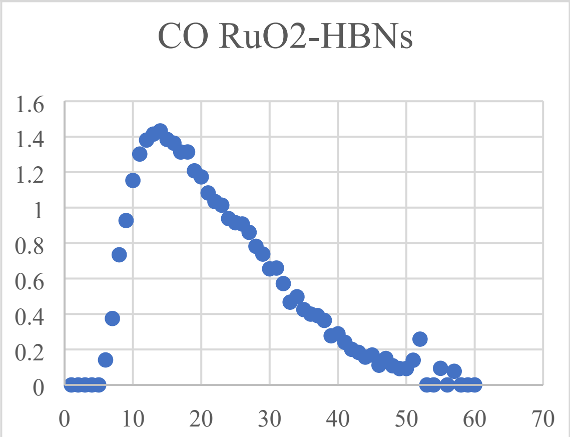
CO RuO2-HBNs
1.6 1.4 1.2 1 0.8 0.6 0.4 0.2 0
0 10 20 30 40 50 60 70

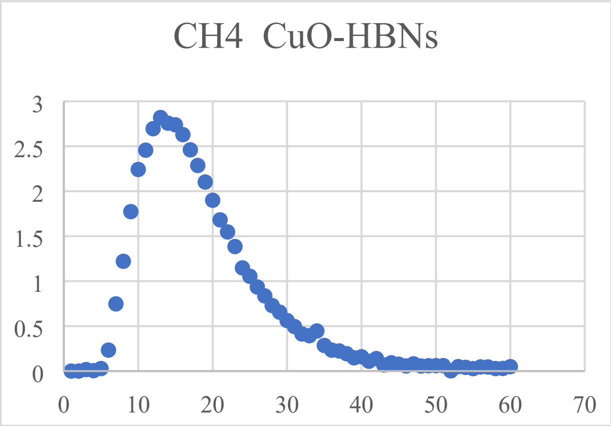
CH4 CuO-HBNs
3 2.5 2 1.5 1 0.5 0
0 10 20 30 40 50 60 70

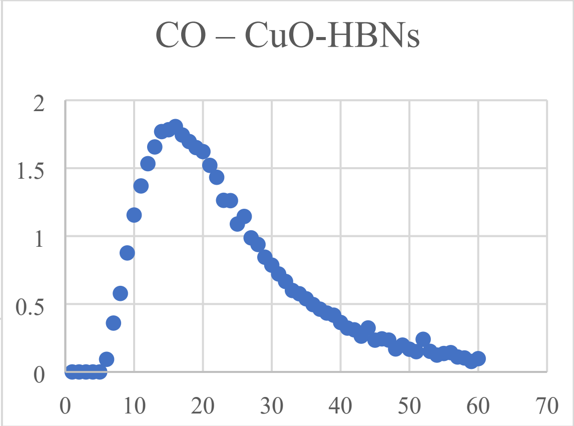
CO – CuO-HBNs
2 1.5 1 0.5 0
0 10 20 30 40 50 60 70

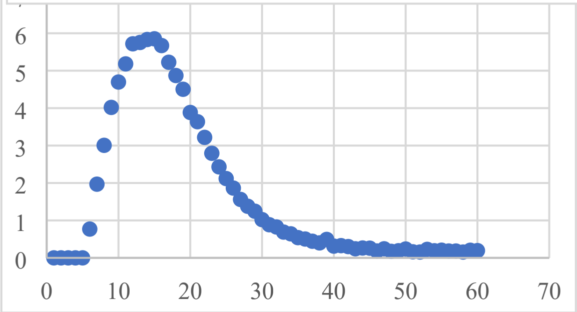
6 5 4 3 2 1 0
0 10 20 30 40 50 60 70

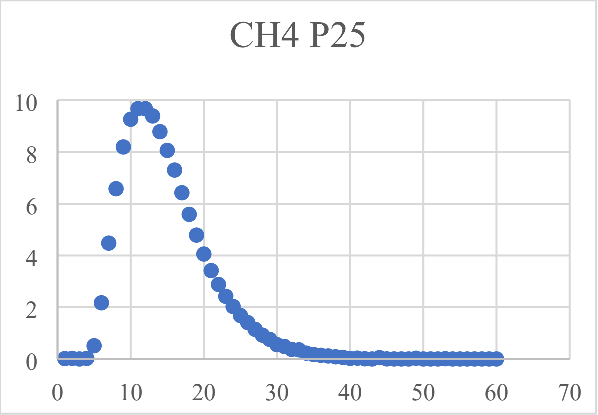

Figure S4 – Gaseous products production (μmols/g_{cat}) plotted against GC cycles for all the examined samples. Each cycle takes 4 minutes to complete, the data above represent 4 hours of reaction.
